# Health effect of agricultural pesticide use in China: implications for the development of GM crops

**DOI:** 10.1038/srep34918

**Published:** 2016-10-10

**Authors:** Chao Zhang, Ruifa Hu, Jikun Huang, Xusheng Huang, Guanming Shi, Yifan Li, Yanhong Yin, Zhaohui Chen

**Affiliations:** 1 Beijing Institute of Technology, 5 South Zhongguancun Street, Beijing 100081, P. R. China; 2 School of Advanced Agricultural Sciences and China Center for Agricultural Policy, Peking University, 5 Yiheyuan Road, Beijing 100871, P. R. China; 3 Department of Neurology, Chinese PLA General Hospital, 28 Fuxing Road, Beijing 100853, P. R. China; 4 Department of Agricultural and Applied Economics, University of Wisconsin-Madison, 427 Lorch Street, Madison, WI 53706, USA; 5 Department of Neurology, the First Affiliated Hospital of PLA General Hospital, No. 51 Fucheng Road, Beijing 100048, P. R. China

## Abstract

It is notable that the adoption of GM glyphosate-tolerant crops increases glyphosate use but reduces non-glyphosate herbicide use; and adoption of GM insect-resistant crops significantly reduces insecticide use. While the health hazard of pesticide use has been well documented, little literature evaluates the health effects of different pesticides related to GM crops in an integrated framework. This study aims to associate the uses of different pesticides related to GM crops with the blood chemistry panel and peripheral nerve conduction of Chinese farmers. Pesticides used by farmers were recorded and classified as glyphosate, non-glyphosate herbicides, chemical lepidopteran insecticides, biological lepidopteran insecticides, non-lepidopteran insecticides and fungicides. The multivariate regression results show that none of the examined 35 health indicators was associated with glyphosate use, while the use of non-glyphosate herbicides was likely to induce renal dysfunction and decrease of serum folic acid. The use of chemical lepidopteran insecticides might be associated with hepatic dysfunction, serum glucose elevation, inflammation and even severe nerve damage. In this context, if GM crops are adopted, the alterations in pesticide use may benefit farmer health in China and globe, which has positive implications for the development of GM crops.

The evaluation report on the carcinogenicity of glyphosate from the International Agency for Research on Cancer (IARC) led to an intensive and immediate debate over the health risks of glyphosate^1^,^2^,^3^,^4^,^5^,^6^. Although much literature argued that glyphosate may not impair human health^7^,^8^,^9^,^10^, or at least glyphosate alone may be harmless^11^,^12^, there is still a growing public concern about the safety of glyphosate use^13^,^14^,^15^,^16^,^17^. With the adoption of the genetically modified (GM) glyphosate-tolerant crops, the share of glyphosate in herbicide uses rise^18^,^19^.

In general, the adoption of GM crops has greatly altered agricultural pesticide use pattern, which is not confined to herbicide use^18^,^19^,^20^,^21^,^22^,^23^,^24^,^25^. As noted, the cultivation of GM glyphosate-tolerant crops has caused a profound alteration in agricultural herbicide use^18^,^19^,^20^. In the United States, for example, the adoption of GM glyphosate-tolerant soybeans induced a 10% decline in the total herbicide use from 1997 to 1998, and 2.5 million kg of glyphosate were used to substitute for 3.3 million kg of non-glyphosate herbicides^25^. In addition, GM insect-resistant crops are associated with the impressive reduction in the use of chemical lepidopteran insecticides since the insecticidal proteins of *Bacillus thuringiensis* produced by GM insect-resistant crops have specific control of lepidopteran insects^26^. In China, the plantation of GM insect-resistant cotton was estimated to reduce insecticide use by 60–80%^27^. Moreover, the adoption of GM insect-resistant maize and cotton was also associated with 136.6 million kg (29.9%) reduction in global insecticide use during the period of 1996 and 2006^28^. In sum, the adoption of GM glyphosate-tolerant crops increases glyphosate use but reduces non-glyphosate herbicide use; and adoption of GM insect-resistant crops significantly reduces insecticide use and change types of insecticides.

Given the above findings on the impacts of GM crops on pesticide use pattern, it is of great importance to evaluate the effects of different pesticides related to GM crops on farmer health. Unfortunately, there is little literature focusing on this issue in an integrated framework. We aim to explore whether the health hazard of glyphosate is severer than that of non-glyphosate herbicides, and how chemical lepidopteran insecticides affect farmer health, both of which have important implications for the development of GM crops in China and globe. For comparison, we also simultaneously assess the health effects of other related groups of pesticides, including biological lepidopteran insecticides, non-lepidopteran insecticides and fungicides.

In contrast with many previous studies, this cohort study focuses on the dose effect of pesticide use on farmer health rather than associate pesticide use with risk ratio, odds ratio and rate difference, which can facilitate observing the sub-clinical health damages of pesticide use but was often ignored. Based on the previous studies, both herbicides and non-herbicide pesticides are likely to be associated with the blood chemistry panel (e.g. hepatic function, renal function and vitamins)^29^,^30^,^31^,^32^,^33^. Moreover, most pesticides (especially organophosphorus chemicals) are neurotoxic substances^34^,^35^,^36^,^37^,^38^,^39^,^40^. However, few studies simultaneously assess the effects of different herbicides (e.g. glyphosate and non-glyphosate herbicides) and non-herbicide pesticides (e.g. lepidopteran insecticides, non-lepidopteran insecticides and fungicides) on these crucial indicators. Accordingly, we examined a wide range of health indicators of the blood chemistry panel and peripheral nerve conduction. The findings from this study can provide references for the further related studies in the future.

## Materials and Methods

### Farmer selection

Farmers in this study were from three Chinese provinces, namely Guangdong, Jiangxi and Hebei, which respectively represent the areas of high, medium and low levels of annual pesticide use in China^41^,^42^. The method of farmer selection was previously described^41^,^43^. In brief, two counties within each province and then two villages within each county were randomly selected. Subsequently, 20–25 farmers were randomly selected from each village. A total of 246 farmers were initially selected to participate in this study, but 22 out of them were excluded because they were absent from the health checks, or failed to provide information on pesticide use. As a result, the final 224 farmers remained. The inclusion criteria for a farmer were as follows: current farmers aged 18 years old and above at the time of farmer selection; and farmers who dominated pesticide use in the household.

This study was approved by the Ethics Committee of Chinese PLA General Hospital. The methods in this study were carried out in accordance with the approved guidelines. The farmer selection was organized by Beijing Institute of Technology. We informed all farmers of the purpose of this study, and obtained informed and written consent before the health checks from all farmers.

### Questionnaire survey

Before the health checks, a face-to-face questionnaire survey was conducted to collect information about the demographic characteristics (e.g. age, gender, education attainment, household population), individual habits (e.g. cigarette intake, alcohol consumption, protective measure when using pesticides), previous pesticide use (e.g. frequency of pesticide use in the past years).

### Health checks

In order to obtain the health information of all farmers, we performed two rounds of health checks in 2012. The first round of health check was performed in March before crop plantation, while the second round was performed in August in Jiangxi and Hebei, and in December in Guangdong, prior to the end of crop harvest. A total of 35 health indicators were examined to describe the blood chemistry panel and peripheral nerve conduction in both rounds of health checks. Note that the indicators of the first round served as the baseline of the analyses. Additionally, the body mass index was calculated for each farmer.

All farmers in this study were asked to fast for no less than 12 hours prior to the blood sample collection. All the blood samples were immediately centrifuged and conserved in a refrigerated condition, and sent to the same laboratory with the same standards in Beijing within no more than 8 hours for chemical test. The related indicators in this study were 13 ones associated with hepatic function, renal function, electrolytes, B vitamins, serum glucose and C-reactive protein (Table S1). In the end, ten farmers failed to fast or provide blood sample.

To measure peripheral nerve conduction, the surface electrodes with standard placement was utilized to perform the conventional nerve conduction studies. The nerve conduction studies were performed on the non-dominant side of each farmer. To ensure the data accuracy, the examined arms and legs of all farmers were warmed using warm water so that the corresponding temperatures were maintained from 32 °C to 34 °C and from 30 °C to 33 °C, respectively. The indicators of the motor and sensory nerve conduction were examined, including the motor conduction velocities, compound muscle action potential amplitudes and distal motor latencies of the median, ulnar, tibial and common peroneal nerves, as well as the sensory conduction velocities and sensory nerve action potential amplitudes of the median, ulnar and sural nerves (Table S2). At last, a total of 218 farmers were included in the analyses of neurotoxicity of pesticide use since we failed to obtain nerve conduction data from six farmers.

### Measuring agricultural pesticide use

In some previous studies, the level of chemical substances or metabolites in human blood or other body fluids was examined to measure pesticide exposure^44^,^45^. However, farmers in this study used many kinds of pesticides. Given our research budget constraints, we had not performed such examinations. As an alternative, we used the actual amount of different pesticides used by farmers as their pesticide exposure.

All farmers were asked to record the detailed information of each pesticide they used. The required information consisted of the chemical name, active ingredient content and actual amount of each pesticide, and the date and hours of each pesticide spraying. To facilitate farmers’ understanding of making the desirable records and ensure the data accuracy and validity, we indeed took some measures: (1) organizing several training sessions to provide some guidelines on the records; (2) revisiting farmers and checking if they made records following our guidelines every other week; and (3) asking farmers to keep the product bottles/bags of all pesticides for the checks.

All pesticides used by farmers were classified into six groups: glyphosate, non-glyphosate herbicides, chemical lepidopteran insecticides, biological lepidopteran insecticides, non-lepidopteran insecticides and fungicides (Table S3). The information of the targeted pests of each pesticide came from China Pesticide Information Network administrated by the Institute for the Control of Agrochemicals, Ministry of Agriculture of China^46^.

### Statistical analysis

To determine the dose effect of pesticide use on farmer health, we conducted a special cohort study rather than by grouping all farmers into exposed and non-exposed groups because nearly all farmers has used pesticides in our samples. Such we could identify what extent each health indicator had changed due to the additional different pesticides used during two rounds of health checks. In order to control the confounding effect of other factors on the health indicators, the multivariate linear regression analyses were utilized and developed as follows:


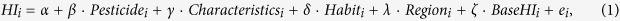


Here, the explained variable *HI*_*i*_ is the indicator of the second round of health check. There are five sets of explanatory variables in equation (1). *Pesticide*_*i*_is a group of variables of pesticide use, including glyphosate, non-glyphosate herbicides, chemical lepidopteran insecticides, biological lepidopteran insecticides, non-lepidopteran insecticides and fungicides. *Characteristics*_*i*_ describes farmers’ demographic characteristics, including age, gender and body mass index. *Habit*_*i*_ is a group of dummy variables of cigarette intake, alcohol consumption and protective measure. *Region*_*i*_ includes two provincial dummies of Guangdong and Jiangxi. *BaseHI*_*i*_ is the indicator of the first round of health check (measuring the baseline health status) corresponding to that of the second round. Since the cumulative duration of pesticide use of each farmer and the related health status before the first round of health check varied across farmers, we included the baseline health variable in the model. Additionally, *e*_*i*_ is the random error term. Moreover, *α*, *β*, *γ*, *δ*, *λ* and *ζ* are the coefficients to be estimated.

Statistical tests were two-sided and only *p* < 0.05 were considered statistically significant. We used STATA version 13.1 (StataCorp LP, College Station, Texas) for all statistical analyses in this study.

## Results

### Demographic characteristics and agricultural pesticide use

The demographic characteristics of farmers are presented in Table 1. The mean age of 224 farmers was about 51.77 years old, and among these farmers were 60 (26.8%) female. The average body mass index was about 23.53 kg/m^2^. Additionally, there were 105 (46.9%) and 95 (42.2%) farmers with habits of cigarette intake and alcohol consumption, respectively. However, only 30 (13.4%) farmers reported that they took protective measure during the pesticide spraying process.

Table 1 also shows the amount of agricultural pesticides used by these farmers in 2012. On average, each farmer used a total of 4.54 kg of pesticides to protect the crops. In terms of herbicide use, the amount of glyphosate was 0.60 kg, almost equalling that of non-glyphosate herbicides (0.61 kg). The average amount of lepidopteran insecticides was 2.38 kg, the majority of which were chemicals (about averagely 2.10 kg), while the average amount of biological lepidopteran insecticides was merely 0.28 kg. The average amount of non-lepidopteran insecticides was 0.27 kg, much lower than that of lepidopteran ones. Compared with herbicides and insecticides, the amount of fungicides used by farmers was only 0.68 kg on average.

### Effects of agricultural pesticide use on the blood chemistry panel

The estimated effects of agricultural pesticide use on the blood chemistry panel were summarized in Table 2. Agricultural glyphosate use was not significantly associated with any indicator of the blood chemistry panel. In contrast, the use of non-glyphosate herbicides was positively associated with two indicators of renal function. Each kilogram increase in non-glyphosate herbicides use inclined to increase blood urea nitrogen (BUN) by 0.10 mmol/L (95% CI: 0.00, 0.19) and creatinine (Cr) by 1.75 *μ*mol/L (95% CI: 0.74, 2.77). We also found that the use of non-glyphosate herbicides showed negative association with serum folic acid (VB_9_) (*β* = −0.35; 95% CI: −0.65, −0.05).

In terms of insecticides, the increase in alanine aminotransferase (ALT) was significantly associated with the use of chemical lepidopteran insecticides (*β* = 0.65; 95% CI: 0.16, 1.14). Additionally, each kilogram increase in chemical lepidopteran insecticides use was also associated with an increase in serum glucose (GLU) of 0.04 mmol/L (95% CI: 0.00, 0.07) and C-reactive protein (CRP) of 0.25 mg/L (95% CI: 0.03, 0.47). However, neither biological lepidopteran nor non-lepidopteran insecticides uses were significantly associated with any indicator of the blood chemistry panel.

In addition, we found that fungicide use was significantly associated with ALT (*β* = 1.43; 95% CI: 0.35, 2.52) and aspartate aminotransferase (AST) (*β* = 1.64; 95% CI: 0.86, 2.41), two crucial indicators of hepatic function. Additionally, each kilogram increase in fungicide use was also associated with a decrease in vitamin B_12_ (VB_12_) of 19.81 ng/L (95% CI: −37.96, −1.66).

### Effects of agricultural pesticide use on the motor nerve conduction

The estimated effects of agricultural pesticide use on the motor nerve conduction were summarized in Table 3. In terms of herbicide use, we found that none of the indicators of the motor nerve conduction had significant association with glyphosate and non-glyphosate herbicides.

We also observed contrasting results of the effects of different types of insecticides on the motor nerve conduction, as presented in Table 3. Agricultural use of chemical lepidopteran insecticides was negatively associated with the motor conduction velocities of the median (MMCV), ulnar (UMCV), tibial (TMCV) and common peroneal (PMCV) nerves. Additionally, each kilogram increase in the use of chemical lepidopteran insecticides was also associated with an increase in the distal motor latency of the ulnar nerve (UDML) of 0.01 ms (95% CI: 0.00, 0.02). However, we found no significant association of the indicators of the motor nerve conduction with the uses of biological lepidopteran and non-lepidopteran insecticides.

In addition, fungicide use was not associated with any indicator of the motor nerve conduction.

### Effects of agricultural pesticide use on the sensory nerve conduction

Table 4 summarized the regression results of the effects of agricultural pesticide use on the sensory nerve conduction. The results showed that none of the indicators of the sensory nerve conduction was significantly associated with the uses of glyphosate and non-glyphosate herbicides. However, each kilogram increase in the use of chemical lepidopteran insecticides was associated with decreases in the sensory conduction velocities of the median (MSCV) of 0.19 m/s (95% CI: −0.38, −0.00) and ulnar (USCV) nerves of 0.20 m/s (95% CI: −0.38, −0.02). In contrast, we also found no significant association of the sensory nerve conduction with biological lepidopteran and non-lepidopteran insecticides uses. Additionally, there was a significantly negative association between fungicide use and the sensory nerve action potential amplitude of ulnar nerve (USNAP) (*β* = −0.20; 95% CI: −0.34, −0.05).

## Discussion

Our results demonstrated that the health effects of different pesticides were different. In general, the adverse health effect of insecticide use was severer than that of the uses of herbicides and fungicides. Glyphosate use was not associated with farmer health damage, while the use of non-glyphosate herbicides inclined to induce renal dysfunction and decrease of serum folic acid. Moreover, chemical lepidopteran insecticides were likely to damage hepatic function, increase serum glucose level and induce the inflammation. It is notable that agricultural use of chemical lepidopteran insecticides might also induce severe damages to peripheral nerves. In contrast, there was not any association between farmer health and the uses of biological lepidopteran and non-lepidopteran insecticides. Additionally, fungicide use probably damaged hepatic function and induced the loss of vitamin B_12_. Given that the adoption of GM glyphosate-tolerant crops can sharply reduce the uses of other herbicides in spite of increasing glyphosate use, and adoption of GM insect-resistant crops can significantly reduce the use of chemical lepidopteran insecticides^18^,^19^,^20^,^21^,^22^,^23^,^24^,^25^, our results implied that the alterations in pesticide use are likely to benefit farmer health if GM crops can be adopted by farmers in China and globe.

Agricultural herbicide use, in this study, was only second to insecticide use, and glyphosate accounted for about half of the total use of herbicides (Table 1). As shown, glyphosate use was not likely to induce negative effect on the blood chemistry panel. It was consistent with the previous studies of human occupational exposure^29^,^30^, but out of accord with the animal studies^47^,^48^,^49^,^50^. It might be attributed to that the relative level of glyphosate intake of the occupational exposed humans was less than that of the oral-administrated experimental animals, and far from enough to induce human health damage. In comparison, the use of non-glyphosate herbicides inclined to increase the risks of renal dysfunction and decrease of serum folic acid. Similar findings were previously published^31^,^32^,^33^. For example, exposures to paraquat, atrazine and acetochlor were associated with renal function damage in both animals and humans^31^,^32^,^33^. Note that these three herbicides were among the most common used ones second to glyphosate in this study. Moreover, we also found that neither glyphosate nor non-glyphosate herbicides uses were associated with peripheral nerve damage.

Agricultural use of lepidopteran insecticides dominated insecticide use in this study, and the majority of lepidopteran insecticides were chemicals (Table 1). Indeed, chemical lepidopteran insecticides mainly consisted of organophosphorous, nereistoxin and pyrethroid insecticides with diffferent health toxicities. The increased ALT, GLU and CRP associated with the use of chemical lepidopteran insecticides demonstrated that substantial use of chemical lepidopteran insecticides was likely to damage hepatic function, increase the serum glucose and induce the inflammation among farmers. The consistent results could also be found in the previous studies^51^,^52^,^53^. For example, organophosphorous insecticides against lepidopteran insects were often associated with sub-clinical hepatotoxicity indicated by the increased serum concentrations of ALT in pregnant women and rats^51^,^52^. In addition, it was well documented that the majority of organophosphorous, nereistoxin and pyrethroid insecticides against lepidopteran insects were nerve agents^34^,^35^,^36^,^37^,^38^,^39^,^40^. Our results showed that the potential damage effects of chemical lepidopteran insecticides on the median and ulnar nerves were far severer.

In contrast with chemical lepidopteran insecticides, biological insecticides against lepidopteran insects were not associated with farmer health risk, which was to be expected. The commonly used biological insecticides against lepidopteran insects in China include *Bacillus thuringiensis* and *Helicoverpa armigera nuclear polyhedrosis virus*. Although these biological lepidopteran insecticides were increasingly used in China due to their high efficiency and low toxicity in crop protection in recent years, the proportion of these insecticides in the total use of lepidopteran insecticides was only about 11.8% (Table 1). In addition, the insecticides used to control non-lepidopteran insects were also not observed to induce adverse effects on farmer health, which could be attributed to that the relative small amount of these insecticides used by farmers was not enough to induce health risk.

Fungicide use accounted for only 15% of the total use of pesticides (Table 1), less than that of insecticides and herbicides. Anyway, we found that fungicide use was likely to damage hepatic function among farmers. Moreover, fungicide use was also associated with the loss of vitamin B_12_. Note that the most common used fungicides in this study consisted of organosulfur chemicals, mainly including ethylenebisdithiocarbamates (EBDCs) and dimethyldithiocarbamates (DMDCs). In detail, among EBDCs were mancozeb, zineb and metiram, and among DMDCs were thiram and ziram. It was previously documented that these organosulfur fungicides were closely associated with the increases in ALT and AST in humans and animals, which indicated damages to hepatic function^54^,^55^,^56^.

It is notable that agricultural uses of glyphosate, non-glyphosate herbicides and lepidopteran insecticides may be greatly altered if GM glyphosate-tolerant and insect-resistant crops are adopted^18^,^19^,^20^,^21^,^22^,^23^,^24^. Although the related increase in glyphosate use has raised several concerns on the health and environmental risks^18^,^20^, substantial studies indicated that glyphosate may be the least toxic pesticide^57^,^58^,^59^,^60^. It implies that the adoption of GM glyphosate-tolerant crops may indeed benefit farmer health by sharply reducing the use of the more toxic non-glyphosate herbicides. In addition, it has also been well-documented that GM insect-resistant crops may enable farmers to protect crops more effectively and thus reduce the use of lepidopteran insecticides, especially chemical ones^21^,^22^,^23^,^24^. In this context, if GM insect-resistant crops are adopted, the alterations in insecticide use may also benefit farmer health by lowering the insecticide exposure level^27^,^61^.

Since 46.9% and 42.4% of farmers in our sample had the habits of cigarette intake and alcohol consumption, respectively, we also conducted statistical tests of the different effects between a group of neither cigarette intake nor alcohol consumption and the other three groups (cigarette intake but not alcohol consumption; alcohol consumption but not cigarette intake; and both cigarette intake and alcohol consumption). However, we did not find evidence that the habits of cigarette intake and alcohol consumption aggravated pesticide intoxication in our sample. This result is not surprising because our study focuses on the sub-clinical health effect rather than acute pesticide intoxication or pesticide intake.

There were also some limitations to be pointed out. The relatively small effective sample size and short study period were likely to produce biased results. In addition, although this study examined a wide range of indicators of the blood chemistry panel and peripheral nerve conduction, it was still hardly reflected the complete health status of those farmers, which then called for further related studies in the future.

In conclusion, glyphosate was likely to be the least toxic to farmer health, while non-glyphosate herbicides inclined to induce hazard effect on farmer renal function and serum folic acid. In terms of insecticides, chemicals used to control lepidopteran insects might cause severe health problems, especially the damages to farmer peripheral nerves, while neither biological lepidopteran nor non-lepidopteran insecticides uses were associated with farmer health. Given the findings on the impacts of GM crops on pesticide use and the results about the health effects of the uses of different pesticides related to GM crops in this study, it can be concluded that the alterations in agricultural pesticide use are likely to benefit the health of a large number of farmers in China and even globe if GM crops are adopted. Hence, this study could have positive implications for the development of GM crops.

## Additional Information

**How to cite this article**: Zhang, C. *et al*. Health effect of agricultural pesticide use in China: implications for the development of GM crops. *Sci. Rep*. **6**, 34918; doi: 10.1038/srep34918 (2016).

## Supplementary Material

Supplementary Information

## Figures and Tables

**Table 1:** Demographic characteristics and agricultural pesticide use (*n* = 224).

Variable	Mean ± Standard Deviation	*n* (%)
Demographic characteristics		
Age (year)	51.77 ± 10.05	
Female (yes = 1, no = 0)		60 (26.8)
Body mass index (kg/m^2^)	23.53 ± 3.64	
Cigarette intake (yes = 1, no = 0)		105 (46.9)
Alcohol consumption (yes = 1, no = 0)		95 (42.4)
Protective measure (yes = 1, no = 0)		30 (13.4)
Agricultural pesticide use (kg)	4.54 ± 5.52	
Glyphosate	0.60 ± 1.57	84 (37.5)
Non-glyphosate herbicides	0.61 ± 1.77	141 (62.9)
Lepidopteran insecticides	2.38 ± 3.44	212 (94.6)
*Chemical insecticides*	2.10 ± 3.41	210 (93.8)
*Biological insecticides*	0.28 ± 0.83	67 (29.9)
Non-lepidopteran insecticides	0.27 ± 0.69	142 (63.4)
Fungicides	0.68 ± 1.56	112 (50.0)

**Table 2:** Effects of pesticide use on the blood chemistry panel (*n* = 214).

Indicator	Glyphosate (kg)	Non-glyphosate herbicides (kg)	Chemical lepidopteran insecticides (kg)	Biological lepidopteran insecticides (kg)	Non-lepidopteran insecticides (kg)	Fungicides (kg)
ALT (U/L)						
*β*	−0.58	−0.05	0.65**	−0.17	−0.55	1.43*
95% CI	−1.68,0.51	−0.88,0.79	0.16,1.14	−2.02,1.69	−2.96,1.85	0.35,2.52
*p*-value	0.29	0.91	<0.01	0.86	0.65	0.01
AST (U/L)						
*β*	−0.06	0.31	0.30	−0.20	0.04	1.64**
95% CI	−0.84,0.71	−0.27,0.90	−0.04,0.65	−1.51,1.11	−1.75,1.82	0.86,2.41
*p*-value	0.87	0.29	0.08	0.77	0.97	0.00
BUN (mmol/L)						
*β*	−0.05	0.10*	0.02	−0.12	−0.23	0.11
95% CI	−0.17,0.07	0.00,0.19	−0.04,0.07	−0.33,0.08	−0.48,0.02	−0.01,0.23
*p*-value	0.39	0.04	0.55	0.24	0.07	0.07
Cr (*μ*mol/L)						
*β*	−0.53	1.75**	−0.21	−0.29	−1.27	0.28
95% CI	−1.87,0.81	0.74,2.77	−0.80,0.38	−2.57,2.00	−4.02,1.48	−1.02,1.59
*p*-value	0.43	0.00	0.49	0.80	0.36	0.67
VB_12_ (ng/L)						
*β*	−0.82	−1.55	−1.02	−6.97	24.10	−19.81*
95% CI	−19.37,17.72	−15.72,12.63	−9.31,7.26	−38.34,24.39	−13.97,62.18	−37.96, −1.66
*p*-value	0.93	0.83	0.81	0.66	0.21	0.03
VB_9_ (*μ*g/L)						
*β*	−0.17	−0.35*	−0.00	0.30	−0.25	0.10
95% CI	−0.57,0.23	−0.65, −0.05	−0.18,0.17	−0.38,0.98	−1.06,0.57	−0.29,0.49
*p*-value	0.40	0.02	0.96	0.39	0.55	0.61
GLU (mmol/L)						
*β*	0.03	0.01	0.04*	0.05	−0.13	−0.01
95% CI	−0.05,0.11	−0.05,0.07	0.00,0.07	−0.08,0.18	−0.29,0.03	−0.08,0.07
*p*-value	0.45	0.77	0.04	0.43	0.11	0.83
CRP (mg/L)						
*β*	−0.24	−0.05	0.25*	0.16	−0.67	0.07
95% CI	−0.73,0.26	−0.43,0.33	0.03,0.47	−0.68,1.00	−1.69,0.35	−0.41,0.56
*p*-value	0.35	0.79	0.03	0.71	0.20	0.77

The result was not reported if none of the coefficient of pesticide variable was statistically significant. The confounders were included but not reported. Please see Tables S4 and S5 for more information. ALT: alanine aminotransferase; AST: aspartate aminotransferase; BUN: blood urea nitrogen; Cr: creatinine; VB_12_: vitamin B_12_; VB_9_: serum folic acid; GLU: serum glucose; CRP: C-reactive protein; CI: confidence interval. **p < 0.01, and *p < 0.05.

**Table 3:** Effects of pesticide use on the motor nerve conduction (*n* = 218).

Indicator	Glyphosate (kg)	Non-glyphosate herbicides (kg)	Chemical lepidopteran insecticides (kg)	Biological lepidopteran insecticides (kg)	Non-lepidopteran insecticides (kg)	Fungicides (kg)
MMCV (m/s)						
*β*	0.18	−0.05	−0.20*	−0.13	0.02	0.28
95% CI	−0.21,0.57	−0.36,0.27	−0.37, −0.02	−0.78,0.52	−0.78,0.82	−0.10,0.66
*p*-value	0.38	0.78	0.03	0.69	0.96	0.15
UMCV (m/s)						
*β*	0.23	−0.14	−0.20*	−0.16	0.25	−0.18
95% CI	−0.16,0.61	−0.45,0.17	−0.37, −0.03	−0.81,0.48	−0.54,1.04	−0.55,0.20
*p*-value	0.25	0.38	0.02	0.62	0.53	0.35
TMCV (m/s)						
*β*	−0.32	−0.11	−0.19*	−0.06	−0.12	−0.05
95% CI	−0.65,0.02	−0.39,0.16	−0.34, −0.04	−0.62,0.50	−0.81,0.57	−0.38,0.28
*p*-value	0.07	0.42	0.01	0.84	0.74	0.77
PMCV (m/s)						
*β*	0.00	−0.04	−0.18**	0.38	−0.34	−0.18
95% CI	−0.30,0.30	−0.28,0.20	−0.31, −0.04	−0.12,0.88	−0.96,0.28	−0.47,0.11
*p*-value	0.99	0.75	<0.01	0.13	0.28	0.23
UDML (ms)						
*β*	−0.01	−0.00	0.01*	−0.05	0.02	0.01
95% CI	−0.03,0.02	−0.03,0.02	0.00,0.02	−0.09,0.00	−0.03,0.08	−0.02,0.03
*p*-value	0.68	0.70	<0.05	0.05	0.39	0.63

The result was not reported if none of the coefficient of pesticide variable was statistically significant. The confounders were included but not reported. Please see Tables S6 and S7 for more information. MMCV: motor conduction velocity of the median nerve; UMCV: motor conduction velocity of the ulnar nerve; TMCV: motor conduction velocity of the tibial nerve; PMCV: motor conduction velocity of the common peroneal nerve; UDML: distal motor latency of the ulnar nerve; CI: confidence interval. **p < 0.01, and *p < 0.05.

**Table 4:** Effects of pesticide use on the sensory nerve conduction (*n* = 218).

Indicator	Glyphosate (kg)	Non-glyphosate herbicides (kg)	Chemical lepidopteran insecticides (kg)	Biological lepidopteran insecticides (kg)	Non-lepidopteran insecticides (kg)	Fungicides (kg)
MSCV (m/s)						
*β*	0.00	−0.34	−0.19*	0.22	0.50	0.08
95% CI	−0.42,0.42	−0.68,0.00	−0.38, −0.00	−0.48,0.92	−0.36,1.36	−0.33,0.48
*p*-value	0.99	0.05	<0.05	0.54	0.26	0.71
USCV (m/s)						
*β*	0.16	−0.04	−0.20*	−0.28	−0.45	−0.16
95% CI	−0.23,0.56	−0.36,0.29	−0.38, −0.02	−0.94,0.37	−1.25,0.36	−0.54,0.22
*p*-value	0.42	0.83	0.03	0.40	0.28	0.42
USNAP (mV)						
*β*	−0.06	0.11	0.04	0.06	0.06	−0.20**
95% CI	−0.20,0.09	−0.01,0.23	−0.02,0.11	−0.18,0.31	−0.24,0.36	−0.34, −0.05
*p*-value	0.45	0.08	0.20	0.60	0.68	<0.01

The result was not reported if none of the coefficient of pesticide variable was statistically significant. The confounders were included but not reported. Please see Table S8 for more information. MSCV: sensory conduction velocity of the median nerve; USCV: sensory conduction velocity of the ulnar nerve; USNAP: sensory nerve action potential amplitude of the ulnar nerve; CI: confidence interval. **p < 0.01, and *p < 0.05.
