# Supplementary material for: Health effect of agricultural pesticide use in China: implications for the development of GM crops
Source: Sci Rep. 2016 Oct 10;6:34918. doi: 10.1038/srep34918 (PMC5056523; doi:10.1038/srep34918)
Supplement: Supplementary Information [file srep34918-s1.pdf]

Supplemental Tables

Health effect of agricultural pesticide use in China: implications for the development of GM crops

Authors:

Chao Zhang, Ruifa Hu, Jikun Huang, Xusheng Huang, Guanming Shi, Yifan Li, Yanhong Yin, and Zhaohui Chen

Table of Contents

Table S1. Definition and description of the indicators of the blood chemistry panel

Table S2. Definition and description of the indicators of peripheral nerve conduction

Table S3. Classification of pesticides used by farmers

Table S4. Effects of pesticide use on the blood chemistry panel (1) (n=214)

Table S5. Effects of pesticide use on the blood chemistry panel (2) (n=214)

Table S6. Effects of pesticide use on motor nerve conduction (1) (n=218)

Table S7. Effects of pesticide use on motor nerve conduction (2) (n=218)

Table S8. Effects of pesticide use on sensory nerve conduction (n=218)

Table S1
Definition and description of the indicators of the blood chemistry panel

Health indicator	Definition	Unit	First round of health check				Second round of health check			
			Mean	SD	Min	Max	Mean	SD	Min	Max
**Hepatic function**										
ALT	Alanine aminotransferase	U/L	24.54	13.45	4.00	106.00	20.18	11.79	4.00	122.00
AST	Aspartate aminotransferase	U/L	26.36	8.91	13.00	72.00	23.01	12.08	12.00	172.00
CHE	Cholinesterase	10^3U/L	8.09	1.66	3.24	12.31	8.71	1.73	4.65	13.53
TP	Total protein	g/L	69.36	3.90	62.00	79.00	76.81	4.84	63.70	92.80
**Renal function**										
BUN	Blood urea nitrogen	mmol/L	5.52	1.42	3.00	11.26	5.15	1.31	1.90	11.40
Cr	Creatinine	μmol/L	75.52	18.07	44.00	122.00	72.99	14.05	44.00	124.00
**Electrolytes**										
Na	Sodium	mmol/L	140.71	2.37	136.16	144.86	141.97	2.49	134.00	151.00
K	Potassium	mmol/L	4.45	0.46	3.60	5.20	4.68	0.67	3.68	9.20
P	Inorganic phosphorus	mmol/L	1.31	0.28	0.88	2.20	1.18	0.15	0.77	1.62
**B vitamins**										
VB_{12}	Vitamin B_{12}	ng/L	480.94	218.30	109.70	945.70	494.93	248.44	142.00	1550.0
VB_9	Serum folic acid	μg/L	10.52	4.04	3.25	19.17	8.29	4.42	2.97	22.00
**GLU**	Serum glucose	mmol/L	4.86	1.00	3.90	14.08	5.46	1.02	4.16	12.48
**CRP**	C-reactive protein	mg/L	1.29	5.01	0.00	52.00	1.62	3.63	0.01	37.20

Data come from authors' survey. SD refers to the standard deviation.

Table S2
Definition and description of the indicators of peripheral nerve conduction

Health indicator	Peripheral nerve name	Unit	First round of health check				Second round of health check			
			Mean	SD	Min	Max	Mean	SD	Min	Max
Motor nerve conduction										
**Motor conduction velocity**										
MMCV	Median nerve	m/s	59.76	4.46	43.50	73.30	58.70	4.25	41.70	68.60
UMCV	Ulnar nerve	m/s	58.00	4.49	46.40	73.70	57.18	4.11	46.40	68.40
TMCV	Tibial nerve	m/s	47.68	3.94	35.90	60.00	47.68	3.85	38.80	63.70
PMCV	Common peroneal nerve	m/s	48.59	4.47	27.00	60.00	48.49	4.32	25.80	57.10
**Proximal compound muscle action potential amplitude**										
MPCMAP	Median nerve	mV	12.20	3.23	5.60	20.90	13.68	3.99	5.70	27.40
UPCMAP	Ulnar nerve	mV	11.33	2.29	5.20	18.90	12.44	2.79	1.00	21.10
TPCMAP	Tibial nerve	mV	11.21	3.86	3.50	25.00	11.21	3.84	3.10	21.40
PPCMAP	Common peroneal nerve	mV	6.50	3.12	0.10	20.20	6.79	3.27	0.10	19.80
**Distal compound muscle action potential amplitude**										
MDCMAP	Median nerve	mV	12.97	3.40	5.60	23.00	14.13	4.11	6.00	28.80
UDCMAP	Ulnar nerve	mV	12.17	2.27	6.10	20.00	13.07	2.71	5.40	23.00
TDCMAP	Tibial nerve	mV	14.31	4.74	4.20	26.70	13.54	4.49	4.30	27.70
PDCMAP	Common peroneal nerve	mV	7.17	3.32	0.10	19.10	7.37	3.34	0.10	18.50
**Distal motor latency**										
MDML	Median nerve	ms	3.34	0.52	2.10	7.20	3.45	0.67	2.60	8.70
UDML	Ulnar nerve	ms	2.63	0.28	2.00	3.60	2.86	0.65	2.00	8.90
TDML	Tibial nerve	ms	3.67	0.46	2.70	4.80	3.69	0.47	2.70	5.00
PDML	Common peroneal nerve	ms	3.73	0.63	2.50	6.70	3.72	0.81	2.70	11.80
Sensory nerve conduction										
**Sensory conduction velocity**										
MSCV	Median nerve	m/s	58.04	6.11	33.30	69.60	58.51	6.33	32.60	72.70
USCV	Ulnar nerve	m/s	54.33	4.53	37.00	65.70	54.17	5.40	20.00	68.00
SSCV	Sural nerve	m/s	59.58	4.98	50.00	75.00	60.44	5.64	50.00	71.40
**Sensory nerve action potential amplitude**										
MSNAP	Median nerve	mV	7.67	3.17	1.20	21.00	8.41	3.35	2.00	21.00
USNAP	Ulnar nerve	mV	5.84	2.49	1.00	17.00	6.29	2.65	0.70	18.00
SSNAP	Sural nerve	mV	15.24	6.91	4.20	44.00	16.22	7.60	4.70	69.00

Data come from authors' survey. SD refers to the standard deviation.

Table S3
Classification of pesticides used by farmers

Pesticide name		No. of spraying farmers	Average amount (kilogram)	
Chinese	English		Per spraying farmers	Per all farmers
**Glyphosate**				
草甘膦	Glyphosate	84	1610.81	604.05
**Non-glyphosate herbicides**				
百草枯	Paraquat	84	379.61	142.36
乙草胺	Acetochlor	23	951.45	97.69
莠去津	Atrazine	11	1376.09	67.58
苯磺隆	Tribenuron-methyl	11	64.16	3.15
乙草胺·莠去津	Acetochlor·Atrazine	9	468.56	18.83
噻吩磺隆	Thifensulfuron-methyl	8	14.49	0.52
苄嘧磺隆·丁草胺	Bensulfuron-methyl·Butachlor	7	47.21	1.48
高效氟吡甲禾灵	Haloxyfop-P-methyl	7	122.89	3.84
异丙草胺·莠去津	Propisochlor·Atrazine	7	757.71	23.68
氟乐灵	Trifluralin	7	1400.86	43.78
甲草胺·乙草胺·莠去津	Alachlor·Acetochlor·Atrazine	6	797.00	21.35
硝磺草酮·莠去津	Mesotrione·Atrazine	6	314.25	8.42
苄嘧磺隆	Bensulfuron-methyl	5	61.32	1.37
草铵膦	Glufosinate-ammonium	5	3800.00	84.82
烟嘧磺隆	Nicosulfuron	5	2760.00	61.61
精喹禾灵	Quizalofop-ethyl	5	10.80	0.24
2,4-滴丁酯	2,4-D butylate	4	31.61	0.56
丁草胺	Butachlor	4	101.40	1.81
丁草胺·异丙草胺·莠去津	Butachlor·Propisochlor·Atrazine	4	434.00	7.75
乙羧氟草醚	Fluoroglycofen-ethyl	4	18.20	0.33
苄嘧磺隆·丙草胺	Bensulfuron-methyl·Pretilachlor	3	43.50	0.58
硝磺草酮	Mesotrione	3	9.33	0.13
甲磺隆	Metsulfuron-methyl	3	411.67	5.51
苄嘧磺隆·乙草胺	Bensulfuron-methyl·Acetochlor	2	14.63	0.13
二甲戊灵·乙草胺	Pendimethalin·Acetochlor	2	80.00	0.71
扑草净·乙草胺	Prometryn·Acetochlor	2	401.00	3.58
乙草胺·莠去津·2,4-滴丁酯	Acetochlor·Atrazine·2,4-D butylate	1	276.48	1.23
甲草胺	Alachlor	1	28.80	0.13
仲丁灵	Butralin	1	144.00	0.64
氰氟草酯	Cyhalofop-butyl	1	20.00	0.09
异丙甲草胺	Metolachlor	1	288.00	1.29
烟嘧磺隆·莠去津	Nicosulfuron·Atrazine	1	60.00	0.27
五氟磺草胺	Penoxsulam	1	5.00	0.02
丙草胺	Pretilachlor	1	90.00	0.40
异丙草胺	Propisochlor	1	130.00	0.58
异丙草胺·乙草胺·莠去津	Propisochlor·Acetochlor·Atrazine	1	520.00	2.32
**Chemical lepidopteran insecticides**				
阿维菌素	Abamectin	119	104.35	55.43
甲维盐	Emamectin benzoate	68	54.81	16.64
氧乐果	Omethoate	40	2257.04	403.04

高效氯氰菊酯	Beta-cypermethrin	38	89.64	15.21
高效氯氟氰菊酯	Lambda-cyhalothrin	38	67.30	11.42
毒死蜱	Chlorpyrifos	35	1281.24	200.19
氯氰菊酯	Cypermethrin	31	133.35	18.46
杀虫双	Bisultap	28	362.39	45.30
甲维盐 丙溴磷	Emamectin benzoate Profenofos	21	130.70	12.25
敌敌畏	DIchlorvos	20	2739.23	244.57
辛硫磷	Phoxim	20	4076.09	363.94
乐果	Dimethoate	17	370.71	28.13
乙酰甲胺磷	Acephate	14	791.14	49.45
高效氯氰菊酯 辛硫磷	Beta-cypermethrin Phoxim	13	311.15	18.06
丁硫克百威	Carbosulfan	13	547.60	31.78
氯氟氰菊酯	Cyhalothrin	13	77.58	4.50
灭多威	Methomyl	13	2170.90	125.99
阿维菌素 啶虫脒	Abamectin Acetamiprid	12	35.16	1.88
联苯菊酯	Bifenthrin	12	18.27	0.98
氟铃脲 毒死蜱	Hexaflumuron Chlorpyrifos	12	94.87	5.08
顺式氯氰菊酯	Alpha-cypermethrin	11	11.02	0.54
阿维菌素 高效氯氰菊酯	Abamectin Beta-cypermethrin	10	28.37	1.27
阿维菌素 毒死蜱	Abamectin Chlorpyrifos	10	620.88	27.72
高效氯氰菊酯 马拉硫磷	Beta-cypermethrin Malathion	10	184.40	8.23
除虫脲	Diflubenzuron	10	728.10	32.50
甲维盐 高效氯氰菊酯	Emamectin benzoate Beta-cypermethrin	10	90.24	4.03
甲维盐 毒死蜱	Emamectin benzoate Chlorpyrifos	10	275.95	12.32
氰戊菊酯	Fenvalerate	10	200.20	8.94
虫酰肼	Tebufenozide	10	2509.70	112.04
氯虫苯甲酰胺	Chlorantraniliprole	9	2329.44	93.59
氰戊菊酯 马拉硫磷	Fenvalerate Malathion	9	98.67	3.96
阿维菌素 甲氰菊酯	Abamectin Fenpropathrin	8	26.34	0.94
溴氰菊酯	Deltamethrin	8	24.55	0.88
甲氰菊酯 氧乐果	Fenpropathrin Omethoate	8	47.06	1.68
氟铃脲	Hexaflumuron	8	32.93	1.18
辛硫磷 马拉硫磷	Phoxim Malathion	8	95.63	3.42
硫丹	Endosulfan	7	984.24	30.76
阿维菌素 灭幼脲	Abamectin Chlorbenzuron	5	314.60	7.02
阿维菌素 氟铃脲	Abamectin Hexaflumuron	5	30.30	0.68
灭幼脲	Chlorbenzuron	5	102.10	2.28
阿维菌素 辛硫磷	Abamectin Phoxim	4	55.25	0.99
克百威	Carbofuron	4	235.00	4.20
硫丹 辛硫磷	Endosulfan Phoxim	4	295.31	5.27
氰戊菊酯 乐果	Fenvalerate Dimethoate	4	211.50	3.78
氟铃脲 辛硫磷	Hexaflumuron Phoxim	4	140.68	2.51
丙溴磷	Profenofos	4	168.00	3.00
敌百虫	Trichlorfon	4	317.50	5.67
高效氯氰菊酯 灭多威	Beta-cypermethrin Methomyl	3	94.50	1.27
氟啶脲	Chlorfuazuron	3	955.00	12.79
氯氰菊酯 毒死蜱	Cypermethrin Chlorpyrifos	3	130.42	1.75
甲维盐 氟铃脲	Emamectin benzoate Hexaflumuron	3	5.99	0.08

氰戊菊酯 敌百虫	Fenvalerate Trichlorfon	3	42.00	0.56
氟虫双酰胺	Fuchongshuangxianan	3	15.10	0.20
阿维菌素 氯氰菊酯	Abamectin Cypermethrin	2	42.50	0.38
阿维菌素 三唑磷	Abamectin Triazophos	2	78.00	0.70
乙酰甲胺磷 毒死蜱	Acephate Chlorpyrifos	2	56.00	0.50
高效氯氰菊酯 氟啶脲	Beta-cypermethrin Chlorfuazuron	2	145.00	1.29
高效氯氰菊酯 毒死蜱	Beta-cypermethrin Chlorpyrifos	2	130.00	1.16
溴氰虫酰胺	Cyantraniliprole	2	16.00	0.14
氯氰菊酯 辛硫磷	Cypermethrin Phoxim	2	16.45	0.15
氯氰菊酯 丙溴磷	Cypermethrin Profenofos	2	66.00	0.59
溴氰菊酯 马拉硫磷	Deltamethrin Malathion	2	143.75	1.28
二嗪磷	Diazinon	2	750.00	6.70
甲维盐 氯氰菊酯	Emamectin benzoate Cypermethrin	2	11.20	0.10
硫丹 灭多威	Endosulfan Methomyl	2	125.00	1.12
醚菊酯	Etofenprox	2	77.50	0.69
甲氰菊酯	Fenpropathrin	2	106.00	0.95
水胺硫磷	Isocarbophos	2	85.00	0.76
高效氯氟氰菊酯 三唑磷	Lambda-cyhalothrin Triazophos	2	975.00	8.71
辛硫磷 灭多威	Phoxim Methomyl	2	402.50	3.59
多杀霉素	Spinosad	2	3.38	0.03
三唑磷	Triazophos	2	46.00	0.41
三唑磷 辛硫磷	Triazophos Phoxim	2	57.00	0.51
阿维菌素 毒死蜱 三唑磷	Abamectin Chlorpyrifos Triazophos	1	30.50	0.14
阿维菌素 氟酰胺	Abamectin Fuchongshuangxianan	1	1.50	0.01
高效氟氯氰菊酯 辛硫磷	Beta-cyfluthrin Phoxim	1	64.50	0.29
高效氯氰菊酯 氟铃脲	Beta-cypermethrin Hexaflumuron	1	6.84	0.03
高效氯氰菊酯 吡虫啉	Beta-cypermethrin Imidacloprid	1	6.00	0.03
高效氯氰菊酯 辛硫磷 马拉硫磷	Beta-cypermethrin Phoxim Malathion	1	1200.00	5.36
高效氯氰菊酯 三唑磷	Beta-cypermethrin Triazophos	1	31.50	0.14
氯虫苯甲酰胺 高效氯氰菊酯	Chlorantraniliprole Beta-cypermethrin	1	35.00	0.16
氯虫苯甲酰胺 高效氯氟氰菊酯	Chlorantraniliprole Lambda-cyhalothrin	1	7.00	0.03
氯氰菊酯 三唑磷	Cypermethrin Triazophos	1	33.00	0.15
甲维盐 啶虫脒	Emamectin benzoate Acetamiprid	1	0.96	0.00
甲维盐 印楝素	Emamectin benzoate Azadirachtin	1	2.40	0.01
甲维盐 氯虫苯甲酰胺	Emamectin benzoate Chlorantraniliprole	1	35.20	0.16
甲维盐 虫酰肼	Emamectin benzoate Tebufenozide	1	48.30	0.22
氰戊菊酯 氧乐果	Fenvalerate Omethoate	1	1125.00	5.02
氰戊菊酯 辛硫磷	Fenvalerate Phoxim	1	48.00	0.21
氰戊菊酯 鱼藤酮	Fenvalerate Rotenone	1	13.65	0.06
茚虫威	Indoxacarb	1	24.00	0.11
高效氯氟氰菊酯 辛硫磷	Lambda-cyhalothrin Phoxim	1	4.80	0.02
氰氟虫腙	Metaflumizone	1	14.40	0.06
杀虫单	Monosultap	1	195.00	0.87
对硫磷	Parathion	1	5.00	0.02
亚胺硫磷	Phosmet	1	120.00	0.54
辛硫磷 毒死蜱	Phoxim Chlorpyrifos	1	100.00	0.45
硫磺 苦参碱	Sulfur Matrine	1	8.19	0.04
特丁硫磷	Terbufos	1	810.00	3.62

噻虫嗪·氯虫苯甲酰胺	Thiamethoxam·Chlorantraniliprole	1	25.60	0.11
**Biological lepidopteran insecticides**				
苏云金杆菌	*Bacillus thuringiensis*	50	622.90	139.04
棉铃虫核型多角体病毒	Heliothis armigera NPV	25	904.80	100.98
苦参碱	Matrine	3	0.65	0.01
斜纹夜蛾核型多角体病毒	Spodopteraliture NPV	3	2740.00	36.70
白僵菌	Beauveria	2	627.50	5.60
印楝素	Azadirachtin	1	1.40	0.01
烟碱·苦参碱	Nicotine·Matrine	1	0.96	0.00
**Non-lepidopteran insecticides**				
啶虫脒	Acetamiprid	85	342.13	129.82
吡虫啉	Imidacloprid	70	127.04	39.70
哒螨灵	Pyridaben	19	26.22	2.22
阿维菌素·哒螨灵	Abamectin·Pyridaben	18	31.72	2.55
马拉硫磷	Malathion	14	943.07	58.94
氟虫腈	Fipronil	10	28.21	1.26
阿维菌素·吡虫啉	Abamectin·Imidacloprid	9	5.49	0.22
噻嗪酮	Buprofezin	6	116.68	3.13
四螨嗪·哒螨灵	Clofentezine·Pyridaben	6	18.65	0.50
灭蝇胺	Cyromazine	6	10.33	0.28
吡虫啉·灭多威	Imidacloprid·Methomyl	5	11.70	0.26
二甲基二硫醚	Dithioether	4	2.50	0.04
异丙威	Isoprocarb	4	26.85	0.48
吡蚜酮	Pymetrozine	4	68.44	1.22
阿维菌素·矿物油	Abamectin·Petroleum oil	3	68.60	0.92
速灭威	Metolcarb	3	84.25	1.13
稻丰散·仲丁威	Phenthoate·Fenobucarb	3	216.00	2.89
甲拌磷	Phorate	3	1548.25	20.74
阿维菌素·炔螨特	Abamectin·Propargite	2	364.00	3.25
噻嗪酮·异丙威	Buprofezin·Isoprocarb	2	33.75	0.30
马拉硫磷·异丙威	Malathion·Isoprocarb	2	8.40	0.08
烯啶虫胺	Nitenpyram	2	55.50	0.50
阿维菌素·四螨嗪	Abamectin·Clofentezine	1	10.40	0.05
阿维菌素·吡蚜酮	Abamectin·Pymetrozine	1	108.00	0.48
啶虫脒·辛硫磷	Acetamiprid·Phoxim	1	60.00	0.27
高效氯氰菊酯·氧乐果	Beta-cypermethrin·Omethoate	1	18.00	0.08
联苯菊酯·啶虫脒	Bifenthrin·Acetamiprid	1	18.00	0.08
噻嗪酮·速灭威	Buprofezin·Metolcarb	1	30.00	0.13
毒杀芬	Camphechlor	1	90.00	0.40
四螨嗪	Clofentezine	1	1.20	0.01
唑螨酯	Fenpyronximate	1	2.25	0.01
氟邦尼	Fubangni	1	82.04	0.37
吡虫啉·仲丁威	Imidacloprid·Fenobucarb	1	240.00	1.07
烯啶虫胺·吡蚜酮	Nitenpyram·Pymetrozine	1	2.40	0.01
甲拌磷·克百威	Phorate·Carbofuron	1	125.00	0.56
吡蚜酮·噻嗪酮	Pymetrozine·Buprofezin	1	35.00	0.16
吡蚜酮·异丙威	Pymetrozine·Isoprocarb	1	12.00	0.05
鱼藤酮	Rotenone	1	1.08	0.00

噻虫嗪	Thiamethoxam	1	7.50	0.03
**Fungicides**				
代森锌	Zineb	35	1248.23	195.04
代森锰锌	Mancozeb	25	903.06	100.79
多菌灵	Carbendazim	22	419.06	41.16
百菌清	Chlorothanonil	20	916.24	81.81
烯酰吗啉	Dimethomorph	17	164.54	12.49
井冈霉素	Jingangmycin	13	50.98	2.96
丙环唑	Propiconazol	13	35.71	2.07
链霉素	Streptomycin	13	58.03	3.37
霜脲氰·代森锰锌	Cymoxanil·Mancozeb	12	607.20	32.53
咪鲜胺	Prochloraz	12	365.93	19.60
苯醚甲环唑	Difenoconazole	11	27.01	1.33
春雷霉素	Kasugamycin	10	3.92	0.18
甲霜灵·代森锰锌	Metalaxyl·Mancozeb	10	494.85	22.09
噁唑菌酮·代森锰锌	Famoxadone·Mancozeb	9	997.64	40.08
吡唑醚菌酯	Pyraclostrobin	9	32.47	1.30
甲基硫菌灵	Thiophanate-methyl	8	273.00	9.75
三唑酮	Triadimefon	8	186.88	6.67
乙蒜素	Ethylicin	7	69.71	2.18
噁霉灵	Hymexazol	7	50.67	1.58
异菌脲	Iprodione	7	56.07	1.75
辛菌胺	Xinjunan	7	10.19	0.32
多菌灵·福美双·溴菌腈	Carbendazim·Thiram·Bromothalonil	6	20.00	0.54
苯醚甲环唑·丙环唑	Difenoconazole·Propiconazol	6	226.90	6.08
烯酰吗啉·吡唑醚菌酯	Dimethomorph·Pyraclostrobin	6	24.15	0.65
盐酸吗啉胍·乙酸铜	Moroxydine hydrochloride·Copper acetate	6	191.00	5.12
霜霉威	Propamocarb	6	216.55	5.80
多菌灵·代森锰锌	Carbendazim·Mancozeb	5	195.00	4.35
烯酰吗啉·代森锰锌	Dimethomorph·Mancozeb	5	86.25	1.93
氢氧化铜	Copper hydroxide	4	67.36	1.20
敌磺钠	Fenaminosulf	4	189.00	3.38
精甲霜灵·代森锰锌	Metalaxyl-M·Mancozeb	4	411.40	7.35
腈菌唑·代森锰锌	Myclobutanil·Mancozeb	4	485.00	8.66
丙森锌	Propineb	4	1369.38	24.45
福美锌	Ziram	4	104.40	1.86
叶枯唑	Bismerthiazol	3	17.67	0.24
多菌灵·福美双	Carbendazim·Thiram	3	22.00	0.29
多菌灵·三唑酮	Carbendazim·Triadimefon	3	115.33	1.54
氯溴异氰尿酸	Chloroisobromine cyanuric acid	3	24.00	0.32
乙酸铜	Copper acetate	3	58.00	0.78
硫酸铜	Copper sulfate	3	71.00	0.95
烯唑醇	Diniconazole	3	27.17	0.36
敌磺钠·福美双	Fenaminosulf·Thiram	3	18.40	0.25
甲霜灵	Metalaxyl	3	48.80	0.65
盐酸吗啉胍·辛菌胺	Moroxydine hydrochloride·Xinjunan	3	4.92	0.07
腈菌唑	Myclobutanil	3	43.73	0.59
咪鲜胺 多菌灵	Prochloraz Carbendazim	3	125.00	1.67

吡唑醚菌酯·代森联	Pyraclostrobin·Metiram	3	240.00	3.21
噻唑锌	Saizuoxin	3	16.00	0.21
噻菌铜	Thiediazole copper	3	19.33	0.26
中生菌素	Zhongshengmycin	3	4.24	0.06
氟吗啉·三乙膦酸铝	Flumorph·Fosetyl-aluminium	2	100.00	0.89
氟硅唑	Flusilazole	2	26.00	0.23
三乙膦酸铝	Fosetyl-aluminium	2	253.50	2.26
三乙膦酸铝·代森锰锌	Fosetyl-aluminium·Mancozeb	2	126.00	1.13
己唑醇	Hexaconazole	2	4.00	0.04
甲霜灵·噁霉灵	Metalaxyl·Hemexazol	2	2.70	0.02
甲霜灵·福美双	Metalaxyl·Thiram	2	1810.00	16.16
代森联	Metiram	2	525.00	4.69
盐酸吗啉胍	Moroxydine hydrochloride	2	155.00	1.38
腈菌唑·福美双	Myclobutanil·Thiram	2	30.00	0.27
宁南霉素	Ningnanmycin	2	5.20	0.05
腐霉利	Procymidone	2	2.75	0.02
松脂酸铜	Songzhisuantong	2	84.70	0.76
福美双·福美锌	Thiram·Ziram	2	72.00	0.64
肟菌酯·戊唑醇	Trifloxystrobin·Tebuconazole	2	28.13	0.25
噻霉酮	Benziothiazolinone	1	4.20	0.02
溴菌腈	Bromothalonil	1	170.00	0.76
溴菌腈·多菌灵	Bromothalonil·Carbendazim	1	100.00	0.45
多菌灵·五氯硝基苯	Carbendazim·Quintozene	1	40.00	0.18
霜脲氰·丙森锌	Cymoxanil·Propineb	1	90.00	0.40
烯酰吗啉·三乙膦酸铝	Dimethomorph·Fosetyl-aluminium	1	500.00	2.23
烯酰吗啉·甲霜灵	Dimethomorph·Metalaxyl	1	6.00	0.03
烯酰吗啉·丙森锌	Dimethomorph·Propineb	1	570.00	2.54
稻瘟净	EBP	1	12.00	0.05
乙嘧酚	Ethirimol	1	420.00	1.88
丁子香酚	Eugenol	1	15.00	0.07
噁唑菌酮·氟硅唑	Famoxadone·Flusilazole	1	51.68	0.23
氟吗啉·代森锰锌	Flumorph·Mancozeb	1	15.00	0.07
香菇多糖	Fungous proteoglycan	1	4.50	0.02
噁霉灵·福美双	Hemexazol·Thiram	1	10.90	0.05
稻瘟灵	Isoprothiolane	1	24.00	0.11
稻瘟灵·己唑醇	Isoprothiolane·Hexaconazole	1	54.00	0.24
精甲霜灵·咯菌腈	Metalaxyl-M·Fludioxonil	1	4.38	0.02
嘧啶核苷类抗菌素	Midingheganleikangjunsu	1	60.00	0.27
噁霜灵·代森锰锌	Oxadixyl·Mancozeb	1	64.00	0.29
喹啉铜	Oxine-copper	1	50.25	0.22
申嗪菌素·丁子香酚·咯菌腈	Phenazino-1-carboxylic acid·Eugenol·Fludioxonil	1	120.00	0.54
多抗霉素	Polyoxin	1	1.20	0.01
咪鲜胺·异菌脲	Prochloraz·Iprodione	1	16.00	0.07
咪鲜胺·松脂酸铜	Prochloraz·Songzhisuantong	1	4.50	0.02
丙森锌·缬霉威	Propineb·Iprovalicarb	1	133.60	0.60
荧光假单胞杆菌	Pseudomonas fluorescens	1	100.00	0.45
嘧霉胺	Pyrimethanil	1	24.00	0.11

噻菌茂	Saijunmao	1	14.00	0.06
戊唑醇	Tebuconazole	1	6.45	0.03
甲基硫菌灵·噁霉灵	Thiophanate-methyl·Hymexazol	1	8.40	0.04
福美双	Thiram	1	75.00	0.33
甲基立枯磷	Tolclofos-methyl	1	16.00	0.07

Table S4
Effects of pesticide use on the blood chemistry panel (1) (*n*=214)

Variables	ALT	AST	CHE	TP	BUN	Cr
Glyphosate	-0.58	-0.06	-0.08	0.02	-0.05	-0.53
	(-1.68,0.51)	(-0.84,0.71)	(-0.18,0.03)	(-0.27,0.30)	(-0.17,0.07)	(-1.87,0.81)
Non-glyphosate herbicides	-0.05	0.31	0.05	0.12	0.10*	1.75**
	(-0.88,0.79)	(-0.27,0.90)	(-0.03,0.13)	(-0.10,0.34)	(0.00,0.19)	(0.74,2.77)
Chemical lepidopteran insecticides	0.65**	0.30	-0.01	0.03	0.02	-0.21
	(0.16,1.14)	(-0.04,0.65)	(-0.06,0.04)	(-0.10,0.15)	(-0.04,0.07)	(-0.80,0.38)
Biological lepidopteran insecticides	-0.17	-0.20	0.10	-0.19	-0.12	-0.29
	(-2.02,1.69)	(-1.51,1.11)	(-0.08,0.27)	(-0.67,0.29)	(-0.33,0.08)	(-2.57,2.00)
Non-lepidopteran insecticides	-0.55	0.04	-0.01	-0.03	-0.23	-1.27
	(-2.96,1.85)	(-1.75,1.82)	(-0.22,0.20)	(-0.62,0.55)	(-0.48,0.02)	(-4.02,1.48)
Fungicides	1.43*	1.64**	-0.02	0.21	0.11	0.28
	(0.35,2.52)	(0.86,2.41)	(-0.12,0.09)	(-0.07,0.48)	(-0.01,0.23)	(-1.02,1.59)
Age (year)	-0.22**	-0.06	-0.00	-0.05*	-0.01	-0.07
	(-0.38,-0.05)	(-0.17,0.05)	(-0.02,0.01)	(-0.09,-0.01)	(-0.03,0.01)	(-0.27,0.13)
Female (yes=1, no=0)	-0.72	-0.03	-0.13	0.75	-0.62**	-13.57**
	(-4.84,3.41)	(-2.93,2.86)	(-0.52,0.27)	(-0.33,1.83)	(-1.08,-0.15)	(-19.65,-7.48)
Body mass index (kg/m2)	1.00**	0.21	0.01	0.10	0.01	0.42
	(0.53,1.46)	(-0.11,0.53)	(-0.04,0.05)	(-0.02,0.22)	(-0.04,0.06)	(-0.15,0.98)
Cigarette intake (yes=1, no=0)	3.05	3.03*	-0.13	-0.99*	-0.14	-3.10
	(-0.51,6.62)	(0.51,5.55)	(-0.47,0.21)	(-1.92,-0.06)	(-0.54,0.26)	(-7.54,1.33)
Alcohol consumption (yes=1, no=0)	-0.35	1.26	-0.03	0.03	-0.06	5.66**
	(-3.62,2.92)	(-1.05,3.58)	(-0.34,0.28)	(-0.82,0.88)	(-0.42,0.30)	(1.66,9.65)
Protective measure (yes=1, no=0)	-2.16	-0.75	0.16	0.40	-0.12	3.05
	(-6.54,2.22)	(-3.85,2.35)	(-0.26,0.58)	(-0.74,1.55)	(-0.60,0.37)	(-2.43,8.54)
Guangdong (yes=1, no=0)	4.70*	3.28*	-0.05	-4.39**	-0.12	2.46
	(0.46,8.95)	(0.28,6.27)	(-0.46,0.36)	(-5.54,-3.25)	(-0.59,0.35)	(-3.18,8.10)
Jiangxi (yes=1, no=0)	4.91*	5.02**	-0.61**	0.18	0.65**	-8.09**
	(0.75,9.07)	(2.07,7.96)	(-1.01,-0.22)	(-0.91,1.26)	(0.19,1.11)	(-13.32,-2.85)
Baseline indicator	0.41**	0.14*	0.70**	0.35**	0.50**	0.36**
	(0.26,0.56)	(0.03,0.25)	(0.61,0.79)	(0.27,0.44)	(0.36,0.63)	(0.18,0.53)
Constant	-1.79	14.91*	2.25**	43.93**	3.12**	47.13**
	(-17.74,14.15)	(3.48,26.35)	(0.67,3.84)	(36.47,51.38)	(1.27,4.97)	(25.50,68.76)
Adjusted R^2	0.36	0.26	0.62	0.48	0.30	0.47

Figures in the parentheses are 95% confidence intervals. ** $p < 0.01$, and * $p < 0.05$.

Table S5

Effects of pesticide use on the blood chemistry panel (2) (*n*=214)

Variables	Na	K	P	VB_{12}	VB_9	GLU	CRP
Glyphosate	0.14	0.04	0.01	-0.82	-0.17	0.03	-0.24
	(-0.10,0.38)	(-0.01,0.09)	(-0.01,0.02)	(-19.37,17.72)	(-0.57,0.23)	(-0.05,0.11)	(-0.73,0.26)
Non-glyphosate herbicides	0.12	0.01	0.00	-1.55	-0.35*	0.01	-0.05
	(-0.06,0.30)	(-0.02,0.05)	(-0.01,0.02)	(-15.72,12.63)	(-0.65,-0.05)	(-0.05,0.07)	(-0.43,0.33)
Chemical lepidopteran insecticides	-0.02	-0.02	0.01	-1.02	-0.00	0.04*	0.25*
	(-0.13,0.08)	(-0.04,0.00)	(-0.00,0.01)	(-9.31,7.26)	(-0.18,0.17)	(0.00,0.07)	(0.03,0.47)
Biological lepidopteran insecticides	-0.19	0.01	-0.00	-6.97	0.30	0.05	0.16
	(-0.59,0.21)	(-0.07,0.09)	(-0.03,0.03)	(-38.34,24.39)	(-0.38,0.98)	(-0.08,0.18)	(-0.68,1.00)
Non-lepidopteran insecticides	0.07	-0.02	0.02	24.10	-0.25	-0.13	-0.67
	(-0.42,0.56)	(-0.12,0.07)	(-0.01,0.06)	(-13.97,62.18)	(-1.06,0.57)	(-0.29,0.03)	(-1.69,0.35)
Fungicides	0.03	-0.02	-0.01	-19.81*	0.10	-0.01	0.07
	(-0.20,0.26)	(-0.06,0.03)	(-0.02,0.01)	(-37.96,-1.66)	(-0.29,0.49)	(-0.08,0.07)	(-0.41,0.56)
Age (year)	0.03	-0.00	0.00	-0.22	-0.03	0.00	-0.01
	(-0.00,0.07)	(-0.01,0.00)	(-0.00,0.00)	(-2.94,2.50)	(-0.09,0.03)	(-0.01,0.01)	(-0.08,0.06)
Female (yes=1, no=0)	0.07	-0.07	0.02	0.03	1.93*	0.10	-0.80
	(-0.83,0.97)	(-0.24,0.10)	(-0.05,0.09)	(-69.30,69.35)	(0.43,3.44)	(-0.19,0.39)	(-2.65,1.06)
Body mass index (kg/m2)	0.06	-0.01	0.00	-3.43	-0.18*	0.00	0.02
	(-0.03,0.16)	(-0.03,0.01)	(-0.00,0.01)	(-11.25,4.38)	(-0.35,-0.02)	(-0.03,0.04)	(-0.19,0.23)
Cigarette intake (yes=1, no=0)	-0.08	0.08	-0.02	-21.17	0.28	-0.03	0.48
	(-0.86,0.69)	(-0.08,0.23)	(-0.07,0.04)	(-81.83,39.48)	(-1.02,1.57)	(-0.28,0.22)	(-1.13,2.10)
Alcohol consumption (yes=1, no=0)	-0.11	-0.07	0.04	21.21	-0.07	0.22	-0.14
	(-0.82,0.60)	(-0.21,0.07)	(-0.02,0.09)	(-34.15,76.57)	(-1.26,1.11)	(-0.01,0.45)	(-1.63,1.34)
Protective measure (yes=1, no=0)	0.77	-0.16	0.03	31.61	1.05	-0.17	-0.80
	(-0.19,1.73)	(-0.35,0.02)	(-0.04,0.09)	(-42.54,105.76)	(-0.54,2.63)	(-0.48,0.14)	(-2.78,1.19)
Guangdong (yes=1, no=0)	-0.46	-0.11	0.49**	-223.64**	-1.59	0.24	-1.04
	(-1.38,0.46)	(-0.29,0.07)	(0.43,0.56)	(-311.40,-135.88)	(-3.34,0.16)	(-0.06,0.54)	(-2.96,0.87)
Jiangxi (yes=1, no=0)	-0.25	-0.07	0.02	43.48	-1.65	-0.20	-1.24
	(-1.20,0.70)	(-0.24,0.11)	(-0.05,0.09)	(-28.59,115.54)	(-3.43,0.13)	(-0.50,0.09)	(-3.13,0.64)
Baseline indicator	0.03	0.06	0.12	-0.02	0.13	0.60**	0.30**
	(-0.12,0.17)	(-0.05,0.16)	(-0.05,0.29)	(-0.15,0.12)	(-0.02,0.29)	(0.49,0.71)	(0.12,0.49)
Constant	134.15**	4.67**	0.89**	653.18**	15.67**	1.27*	1.44
	(112.82,155.47)	(3.88,5.45)	(0.57,1.21)	(381.68,924.69)	(9.88,21.47)	(0.09,2.44)	(-5.83,8.71)
Adjusted R^2	0.02	0.00	0.63	0.30	0.06	0.42	0.04

Figures in the parentheses are 95% confidence intervals. ** $p < 0.01$, and * $p < 0.05$.

Table S6

Effects of pesticide use on motor nerve conduction (1) (n=218)

Variables	MMCV	UMCV	TMCV	PMCV	MDML	UDML	TDML	PDML
Glyphosate	0.18	0.23	-0.32	0.00	0.01	-0.01	0.04	0.03
	(-0.21,0.57)	(-0.16,0.61)	(-0.65,0.02)	(-0.30,0.30)	(-0.04,0.06)	(-0.03,0.02)	(-0.00,0.09)	(-0.02,0.07)
Non-glyphosate herbicides	-0.05	-0.14	-0.11	-0.04	-0.00	-0.00	-0.01	-0.00
	(-0.37,0.27)	(-0.45,0.17)	(-0.39,0.16)	(-0.28,0.20)	(-0.04,0.04)	(-0.03,0.02)	(-0.05,0.03)	(-0.04,0.03)
Chemical lepidopteran insecticides	-0.20*	-0.20*	-0.19*	-0.18**	0.02	0.01*	-0.01	-0.01
	(-0.37,-0.02)	(-0.38,-0.03)	(-0.34,-0.04)	(-0.31,-0.04)	(-0.00,0.04)	(0.00,0.02)	(-0.03,0.01)	(-0.03,0.01)
Biological lepidopteran insecticides	-0.13	-0.16	-0.06	0.38	0.01	-0.05	0.01	0.02
	(-0.78,0.52)	(-0.81,0.48)	(-0.62,0.50)	(-0.12,0.88)	(-0.07,0.09)	(-0.09,0.00)	(-0.07,0.08)	(-0.06,0.09)
Non-lepidopteran insecticides	0.02	0.25	-0.12	-0.34	-0.04	0.02	0.01	0.08
	(-0.78,0.82)	(-0.54,1.04)	(-0.81,0.57)	(-0.96,0.28)	(-0.14,0.06)	(-0.03,0.08)	(-0.08,0.10)	(-0.02,0.17)
Fungicides	0.28	-0.18	-0.05	-0.18	-0.00	0.01	0.01	0.02
	(-0.10,0.66)	(-0.55,0.20)	(-0.38,0.28)	(-0.47,0.11)	(-0.05,0.05)	(-0.02,0.03)	(-0.03,0.05)	(-0.03,0.06)
Age (year)	-0.09**	-0.07*	-0.03	-0.03	0.01	0.00*	-0.00	0.00
	(-0.15,-0.03)	(-0.13,-0.01)	(-0.08,0.02)	(-0.08,0.01)	(-0.00,0.01)	(0.00,0.01)	(-0.01,0.00)	(-0.00,0.01)
Female (yes=1, no=0)	-0.81	1.65*	-0.65	0.61	-0.01	-0.11*	-0.23**	0.01
	(-2.28,0.67)	(0.20,3.11)	(-1.90,0.60)	(-0.51,1.73)	(-0.19,0.17)	(-0.22,-0.01)	(-0.39,-0.06)	(-0.16,0.18)
Body mass index (kg/m2)	-0.07	-0.04	-0.03	0.03	0.02	-0.01	-0.00	-0.01
	(-0.24,0.11)	(-0.21,0.13)	(-0.18,0.12)	(-0.10,0.17)	(-0.01,0.04)	(-0.02,0.00)	(-0.02,0.02)	(-0.03,0.01)
Cigarette intake (yes=1, no=0)	-0.62	0.44	-0.25	-0.28	-0.07	-0.08	-0.12	-0.01
	(-1.88,0.63)	(-0.81,1.69)	(-1.33,0.83)	(-1.24,0.69)	(-0.22,0.09)	(-0.17,0.00)	(-0.26,0.02)	(-0.15,0.14)
Alcohol consumption (yes=1, no=0)	0.07	-0.35	-0.87	-0.20	-0.05	0.02	-0.02	0.08
	(-1.08,1.22)	(-1.48,0.79)	(-1.86,0.12)	(-1.08,0.68)	(-0.20,0.09)	(-0.06,0.10)	(-0.15,0.11)	(-0.05,0.22)
Protective measure (yes=1, no=0)	0.35	0.03	0.25	0.57	-0.18	0.01	0.13	0.14
	(-1.27,1.97)	(-1.57,1.63)	(-1.14,1.65)	(-0.68,1.82)	(-0.38,0.02)	(-0.11,0.12)	(-0.05,0.31)	(-0.05,0.33)
Guangdong (yes=1, no=0)	0.83	0.44	0.66	0.29	0.04	0.12*	-0.16	-0.19*
	(-0.68,2.34)	(-1.05,1.93)	(-0.64,1.96)	(-0.87,1.45)	(-0.15,0.23)	(0.01,0.22)	(-0.33,0.01)	(-0.37,-0.02)
Jiangxi (yes=1, no=0)	0.69	0.56	-0.24	-0.30	-0.07	0.02	0.03	-0.02
	(-0.80,2.17)	(-0.92,2.04)	(-1.51,1.03)	(-1.43,0.83)	(-0.25,0.11)	(-0.09,0.12)	(-0.14,0.19)	(-0.19,0.16)
Baseline indicator	0.40**	0.45**	0.40**	0.67**	0.27**	0.01	0.33**	0.52**
	(0.27,0.53)	(0.31,0.59)	(0.28,0.53)	(0.57,0.77)	(0.17,0.37)	(-0.05,0.07)	(0.19,0.46)	(0.44,0.60)
Constant	42.62**	36.34**	32.15**	17.47**	1.76**	2.57**	2.79**	1.87**
	(32.16,53.08)	(25.74,46.94)	(23.86,40.43)	(10.27,24.67)	(0.97,2.54)	(2.12,3.02)	(1.95,3.62)	(1.13,2.60)
Adjusted R^2	0.25	0.27	0.28	0.56	0.16	0.09	0.11	0.48

Figures in the parentheses are 95% confidence intervals. ** $p < 0.01$, and * $p < 0.05$.

Table S7

Effects of pesticide use on motor nerve conduction (2) (*n*=218)

Variables	MPCMAP	MDCMAP	UPCMAP	UDCMAP	TPCMAP	TDCMAP	PPCMAP	PDCMAP
Glyphosate	0.22	0.24	0.01	0.03	0.06	-0.11	0.01	0.06
	(-0.04,0.48)	(-0.03,0.51)	(-0.16,0.18)	(-0.13,0.19)	(-0.18,0.30)	(-0.42,0.20)	(-0.17,0.18)	(-0.13,0.25)
Non-glyphosate herbicides	0.05	0.04	0.03	0.01	-0.01	0.05	0.05	0.12
	(-0.16,0.26)	(-0.17,0.26)	(-0.11,0.17)	(-0.12,0.14)	(-0.20,0.18)	(-0.20,0.31)	(-0.10,0.19)	(-0.04,0.27)
Chemical lepidopteran insecticides	-0.05	-0.05	0.00	-0.00	-0.11	-0.09	0.00	-0.01
	(-0.16,0.07)	(-0.17,0.07)	(-0.07,0.08)	(-0.07,0.07)	(-0.21,0.00)	(-0.23,0.05)	(-0.08,0.08)	(-0.10,0.07)
Biological lepidopteran insecticides	-0.06	-0.10	0.12	0.08	-0.04	-0.19	-0.04	-0.05
	(-0.50,0.37)	(-0.54,0.35)	(-0.17,0.41)	(-0.19,0.35)	(-0.44,0.36)	(-0.70,0.33)	(-0.33,0.25)	(-0.36,0.27)
Non-lepidopteran insecticides	-0.16	-0.19	0.07	-0.06	-0.26	-0.51	-0.31	0.03
	(-0.69,0.37)	(-0.73,0.36)	(-0.28,0.41)	(-0.38,0.27)	(-0.76,0.23)	(-1.15,0.13)	(-0.67,0.05)	(-0.36,0.41)
Fungicides	-0.24	-0.20	-0.06	-0.06	0.18	0.28	-0.05	-0.08
	(-0.49,0.01)	(-0.46,0.06)	(-0.22,0.10)	(-0.21,0.10)	(-0.05,0.41)	(-0.02,0.58)	(-0.22,0.12)	(-0.26,0.10)
Age (year)	-0.02	-0.03	-0.02	-0.01	-0.07**	-0.06*	-0.03*	-0.05**
	(-0.06,0.02)	(-0.07,0.01)	(-0.04,0.01)	(-0.03,0.01)	(-0.10,-0.03)	(-0.11,-0.01)	(-0.06,-0.01)	(-0.08,-0.02)
Female (yes=1, no=0)	-0.42	-0.60	0.28	0.10	0.54	1.05	0.38	0.31
	(-1.39,0.55)	(-1.59,0.39)	(-0.36,0.92)	(-0.50,0.71)	(-0.35,1.42)	(-0.10,2.20)	(-0.28,1.04)	(-0.40,1.02)
Body mass index (kg/m2)	0.02	0.02	-0.03	0.00	-0.09	0.01	-0.08*	-0.03
	(-0.09,0.14)	(-0.10,0.13)	(-0.11,0.04)	(-0.07,0.07)	(-0.20,0.02)	(-0.12,0.15)	(-0.16,-0.01)	(-0.11,0.06)
Cigarette intake (yes=1, no=0)	-0.46	-0.73	-0.15	0.03	0.02	0.04	0.20	-0.04
	(-1.29,0.38)	(-1.59,0.12)	(-0.70,0.40)	(-0.49,0.54)	(-0.74,0.78)	(-0.96,1.03)	(-0.36,0.77)	(-0.65,0.57)
Alcohol consumption (yes=1, no=0)	0.03	0.16	0.15	0.08	-0.39	0.07	-0.34	-0.11
	(-0.73,0.80)	(-0.62,0.94)	(-0.34,0.65)	(-0.39,0.55)	(-1.09,0.31)	(-0.84,0.98)	(-0.85,0.18)	(-0.66,0.45)
Protective measure (yes=1, no=0)	0.57	0.28	0.56	0.39	1.22*	0.21	0.16	-0.34
	(-0.52,1.65)	(-0.83,1.38)	(-0.14,1.27)	(-0.27,1.05)	(0.23,2.20)	(-1.08,1.50)	(-0.57,0.89)	(-1.13,0.45)
Guangdong (yes=1, no=0)	0.56	0.58	0.31	0.44	-1.31**	-1.62**	0.40	0.49
	(-0.44,1.57)	(-0.44,1.61)	(-0.35,0.97)	(-0.18,1.06)	(-2.23,-0.39)	(-2.82,-0.43)	(-0.27,1.08)	(-0.24,1.22)
Jiangxi (yes=1, no=0)	-0.11	-0.53	-1.16**	-1.07**	-1.72**	-1.74**	-0.74*	-0.96**
	(-1.09,0.86)	(-1.53,0.47)	(-1.80,-0.53)	(-1.67,-0.47)	(-2.62,-0.82)	(-2.91,-0.58)	(-1.40,-0.08)	(-1.67,-0.25)
Baseline indicator	0.50**	0.51**	0.47**	0.52**	0.68**	0.74**	0.74**	0.75**
	(0.40,0.59)	(0.42,0.61)	(0.38,0.56)	(0.44,0.61)	(0.59,0.77)	(0.64,0.84)	(0.66,0.82)	(0.66,0.83)
Constant	6.09**	7.22**	7.26**	5.75**	10.15**	7.94**	5.35**	4.93**
	(1.66,10.52)	(2.67,11.77)	(4.44,10.07)	(3.07,8.43)	(6.12,14.17)	(2.73,13.14)	(2.44,8.25)	(1.75,8.10)
Adjusted R^2	0.36	0.40	0.46	0.51	0.63	0.58	0.69	0.68

Figures in the parentheses are 95% confidence intervals. ** $p < 0.01$, and * $p < 0.05$.

Table S8
Effects of pesticide use on sensory nerve conduction (*n*=218)

Variables	MSCV	USCV	SSCV	MSNAP	USNAP	SSNAP
Glyphosate	0.00	0.16	-0.10	-0.12	-0.06	-0.15
	(-0.42,0.42)	(-0.23,0.56)	(-0.55,0.36)	(-0.32,0.09)	(-0.20,0.09)	(-0.64,0.35)
Non-glyphosate herbicides	-0.34	-0.04	-0.13	0.08	0.11	-0.15
	(-0.68,0.00)	(-0.36,0.29)	(-0.49,0.24)	(-0.08,0.25)	(-0.01,0.23)	(-0.55,0.25)
Chemical lepidopteran insecticides	-0.19*	-0.20*	0.01	-0.00	0.04	0.05
	(-0.38,-0.00)	(-0.38,-0.02)	(-0.20,0.21)	(-0.10,0.09)	(-0.02,0.11)	(-0.17,0.27)
Biological lepidopteran insecticides	0.22	-0.28	-0.30	-0.04	0.06	-0.34
	(-0.48,0.92)	(-0.94,0.37)	(-1.05,0.46)	(-0.39,0.30)	(-0.18,0.31)	(-1.17,0.48)
Non-lepidopteran insecticides	0.50	-0.45	-0.53	-0.13	0.06	0.41
	(-0.36,1.36)	(-1.25,0.36)	(-1.46,0.40)	(-0.56,0.29)	(-0.24,0.36)	(-0.61,1.42)
Fungicides	0.08	-0.16	-0.23	-0.02	-0.20**	-0.42
	(-0.33,0.48)	(-0.54,0.22)	(-0.67,0.21)	(-0.22,0.18)	(-0.34,-0.05)	(-0.90,0.06)
Age (year)	-0.09**	-0.16**	-0.05	-0.05**	-0.07**	-0.18**
	(-0.15,-0.03)	(-0.22,-0.10)	(-0.12,0.02)	(-0.08,-0.02)	(-0.09,-0.05)	(-0.26,-0.10)
Female (yes=1, no=0)	-0.75	1.43	1.75*	0.06	0.68*	-0.28
	(-2.31,0.81)	(-0.04,2.90)	(0.04,3.46)	(-0.71,0.83)	(0.11,1.25)	(-2.12,1.56)
Body mass index (kg/m2)	-0.24*	0.04	0.16	0.01	-0.05	-0.26*
	(-0.42,-0.05)	(-0.13,0.22)	(-0.04,0.36)	(-0.09,0.10)	(-0.12,0.01)	(-0.48,-0.04)
Cigarette intake (yes=1, no=0)	0.27	0.12	-0.34	-0.34	0.30	-0.74
	(-1.08,1.62)	(-1.15,1.38)	(-1.80,1.11)	(-1.00,0.32)	(-0.17,0.77)	(-2.33,0.85)
Alcohol consumption (yes=1, no=0)	0.14	0.51	-0.69	0.25	-0.21	-0.28
	(-1.10,1.37)	(-0.65,1.67)	(-2.02,0.64)	(-0.36,0.85)	(-0.65,0.22)	(-1.73,1.18)
Protective measure (yes=1, no=0)	-0.97	-0.90	-1.38	0.22	0.36	0.11
	(-2.73,0.80)	(-2.53,0.74)	(-3.28,0.52)	(-0.64,1.07)	(-0.24,0.97)	(-1.95,2.17)
Guangdong (yes=1, no=0)	-2.04*	-0.12	-0.45	-0.24	-0.02	0.89
	(-3.66,-0.41)	(-1.64,1.39)	(-2.20,1.31)	(-1.05,0.56)	(-0.59,0.55)	(-1.03,2.81)
Jiangxi (yes=1, no=0)	2.67**	0.56	-0.88	0.02	0.12	-0.01
	(1.08,4.26)	(-1.00,2.11)	(-2.60,0.85)	(-0.76,0.80)	(-0.44,0.67)	(-1.88,1.86)
Baseline indicator	0.63**	0.25**	0.25**	0.68**	0.63**	0.50**
	(0.53,0.72)	(0.14,0.36)	(0.14,0.37)	(0.59,0.77)	(0.55,0.72)	(0.40,0.60)
Constant	31.89**	47.87**	44.17**	4.44**	6.25**	23.18**
	(22.80,40.98)	(39.35,56.39)	(34.54,53.80)	(1.17,7.70)	(3.84,8.67)	(15.19,31.18)
Adjusted R^2	0.54	0.26	0.19	0.58	0.66	0.50

Figures in the parentheses are 95% confidence intervals. ** $p < 0.01$, and * $p < 0.05$.
